# Work-related disease symptoms and occupational injuries among coffee processing industry workers in Bench-Sheko and Kaffa Zones Southwest, Ethiopia: A mixed-method study

**DOI:** 10.3389/fpubh.2022.1034957

**Published:** 2022-12-22

**Authors:** Besufekad Mekonnen, Nahom Solomon, Wondimagegn Wondimu, Melkamsew Tesfaye, Samuel Negash

**Affiliations:** ^1^Department of Environmental Health, College of Medicine and Health Sciences, Mizan-Tepi University, Mizan-Aman, Ethiopia; ^2^Department of Public Health, College of Medicine and Health Sciences, Mizan-Tepi University, Mizan-Aman, Ethiopia

**Keywords:** work-related disease symptoms, occupational injuries, coffee processing industries, Southwest Ethiopia, work-related symptoms

## Abstract

**Background:**

Occupational injuries have become one of the most critical rooting causes paying to infirmities and life-threatening conditions in developed and developing countries. Workers in the coffee industry face some occupational health and safety issues. However, there is limited evidence on this important public health issue. Hence, this research was conducted to assess work-related disease symptoms and occupational injuries among coffee processing workers in Southwest, Ethiopia.

**Methods:**

A cross-sectional study supplemented with a qualitative method was done. A total of 721 workers were involved in the study for quantitative information. In addition, we performed a total of 22 in-depth and five key informant interviews for generating qualitative evidence. Quantitative data was collected by an interview-based questionnaire which is adapted from similar studies. We conducted descriptive, binary logistic, and multivariable regression analysis as necessary, to ascertain the factors affecting occupational injuries. We collected qualitative data guided by an interview guide, transcribed verbatim, and analyzed using ATLAS ti version-8 by applying a content analysis approach. Finally, quotes from participants that had exemplary ideas were triangulated along with quantitative findings.

**Result:**

The overall prevalence rate of work-related symptoms and occupational injuries among coffee processing workers were 21.7 and 13.4% respectively. Age group 30–39 and 40–49 (Adjusted odds ratio (AOR) 1.95, 95% CI 1.37, 2.79, (AOR 3.28, 95% CI 1.89, 5.69, respectively, income level (AOR 0.24, 95% CI 0.16, 0.36, *p* = 0.000), experience (AOR 1.64, 95% CI 1.04, 2.60, *p* = 0.034), and smoking cigarette (AOR 5.59, 95% CI 2.78, 11.26, *p* = 0.000) were significantly associated with the work-related symptom. In addition, training related to the job (AOR 11.88, 95% CI1.34, 105.57, *p* = 0.026) was significantly associated with occupational injuries among coffee processing industry workers.

**Conclusion:**

The prevalence of work-related symptoms and occupational injuries was high among coffee processing industry workers in southwest Ethiopia. Therefore, there is a need for regulations for both government and industry owners to advance the occupational conditions and ergonomic structure of coffee processing industries.

## 1. Introduction

Job-related injuries are one of the largest reasons contributing to disabilities and life-threatening conditions in developed and developing countries (1–4). Occupational diseases existing a main public health issue resulting in serious social and economic problem that could be prevented if appropriate measures are taken (5, 6). Globally, an estimated 271 million people suffer from occupational injuries, and 2 million die each year as a result of these work-related injuries (7–9).

The estimated economic loss triggered by occupational accidents and disease was equivalent to 4% of the world's gross national product (10). According to International Labor Organization, 2.3 million workers die each year from unintended job-related accidents and diseases. Individuals belonging to all economic groups hurt fatal injuries, but death rates due to injury tend to be higher in those from developing countries where there is an insecure working environment and less awareness (11, 12).

In Ethiopia, the manufacturing sector and working areas are growing alarmingly. As a result, the problem of injury is severe due to the lack of a healthy working environment in the rate of industrial expansion (1, 13, 14). Moreover, only 5–10% of the workforce in Ethiopia have access to some kind of work-related health services and trained workers, limited/no job-related services and psychosocial stress are exist (15, 16).

Currently, 335/1,000 workers are exposed to occupational injury per year in small and medium-scale industries. Of these, 17.1% of them were hospitalized, 40% of them for greater than 24 h, 53.9% were absent from work, and 191 days were lost due to work-related injuries [10. 16]. Another study in Afar prevailed that the overall prevalence rate was 783 per 1,000 workers with 11% being hospitalized and 153 days lost due to injuries (17).

Studies in primary coffee processing factories in Uganda and Sri Lanka have indicated a higher prevalence of acute respiratory symptoms than among controls. Similarly, an increased prevalence of chronic respiratory symptoms has been reported among primary coffee factory workers in Papua New Guinea and Tanzania (18–20).

Ethiopia is a major producer of coffee in Africa producing about 500,000 tones every year (21). Ethiopia is the birthplace of Coffea arabica, which obtained its name from Kaffa where coffee was first discovered in the southwestern highlands of Ethiopia. Coffee contributes to about 10% of the Ethiopian gross domestic product and accounts for more than 25% of the foreign currency income (20, 22). In Ethiopia, about 15 million people depend on coffee production directly or indirectly for their living (23).

Ethiopia is one of the 10 top coffee producers in the world. According to the International coffee organization (ICO), Ethiopia was the fifth largest coffee producer after Brazil, Vietnam, Indonesia, and Colombia, with a total production of 498,780 tones; and the seventh largest coffee exporter in the world (20, 22, 24).

According to the Ethiopian Government figures, in 2019/2020 the total volume and value of Ethiopian's coffee export were 196,117 tones and 841.65 million American Dollars respectively. This volume and value of coffee export when compared to the 2018/19 export performance shows increment was increased by 13.9 and 59.3%, respectively (25).

Despite all these facts; in Ethiopia, there is a lack of wide-ranging data and nationwide research on the rate of work-related disease symptoms and occupational injuries and its factors in coffee processing industry workers. Thus, this study aimed to assess work-related disease symptoms, occupational injuries, and associated factors among coffee processing workers in the Bench-Sheko and Kaffa Zones, in southwest Ethiopia.

## 2. Methods

### 2.1. Study area and period

**Figure 1:**
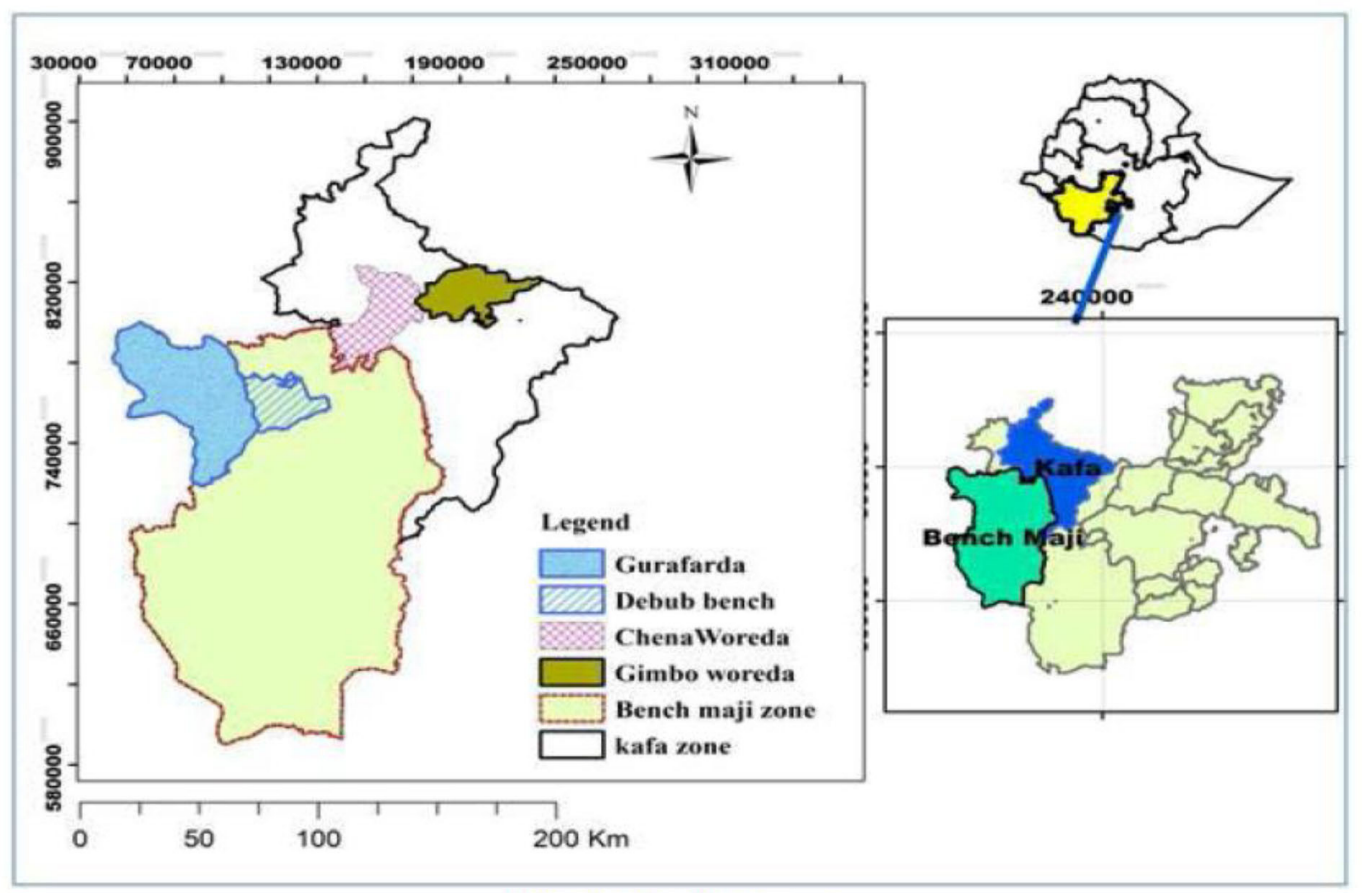
Map of study area.

The study was conducted in the Bench-Sheko and Kaffa Zones from February 15 up to June 30, 2021 (Figure 1). These two zones are among the high coffee-producing districts in Ethiopia. Their capital towns (Mizan-Aman and Bonga) are at 585 km and 469 km respectively from Addis Ababa, the capital of Ethiopia, to the southwest direction.

According to reports from the zones' industry offices; there are a total of 175 functional coffee processing industries in these zones. Of these 82 (53 for wet coffee and 29 for dry coffee) are found in the Bench-Sheko zone and 93 (58 for spike coffee and 35 for dry coffee) are found in the Kaffa zone. The number of workers in these coffee processing industries varies depending on the season of coffee harvesting. The number of workers in these industries varies with time. It increases in the harvesting season particularly from September to May; since temporary employees are involved in coffee processing. And it decreases in the other seasons.

### 2.2. Study design

An institution-based cross-sectional study design supplemented by a qualitative study was conducted.

### 2.3. Populations

#### 2.3.1. Source population

All of the workers in coffee processing industries found in the Bench-Sheko and Kaffa zones were the source population.

#### 2.3.2. Study population

All workers in the selected coffee processing industries found in the Bench-Sheko and Kaffa zones were the study population.

### 2.4. Eligibility criteria

#### 2.4.1. Inclusion criteria

Workers who are employed and being on job for at least 6 months were included.

#### 2.4.2. Exclusion criteria

Administrative workers were not included (for the quantitative study) because they may not be involved directly in tasks that put them at risk for occupational injuries. Besides, workers who had chronic illnesses and who had a previous history of musculoskeletal health problems not related to their occupation were excluded from the study.

### 2.5. Sampling size and sampling techniques

#### 2.5.1. Sampling size determination

##### 2.5.1.1. For quantitative study

**Figure 2:**
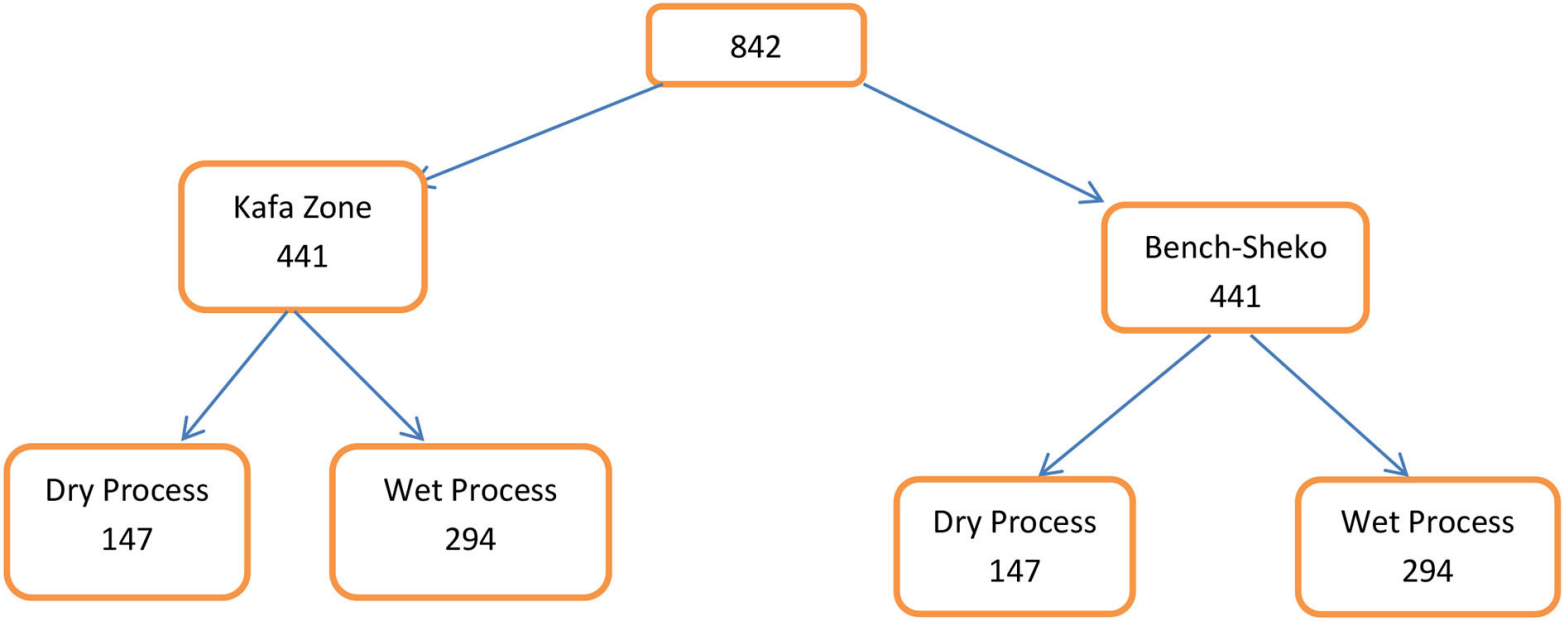
The schematic presentation of the sampling process.

The sample size was calculated using a single population proportion formula taking the prevalence of cough among coffee processing industries workers in Ethiopia which were 46.4% (25). In addition to this, taking a confidence level of 95%, a margin of error of 5%, and a design effect of 2, the sample size became 765. But considering a non-response rate of 10%, about 842 workers were considered (Figure 2) to be included in the study, even though we received a response from only 721 workers.

##### 2.5.1.2. For qualitative study

In-depth interviews with 22 purposively selected workers and five key informant interviews were conducted based on the saturation of the information.

#### 2.5.2. Sampling technique

##### 2.5.2.1. For quantitative study

First, among the total coffee processing industries found in both zones, 30% of each type of coffee processing industry were selected randomly from each zone. Then, allocating proportionally, the final sample was selected by a systematic random sampling technique using the list of workers, which was obtained from the registries of industries, as a sampling frame.

##### 2.5.2.2. For qualitative study

A purposive sampling technique was employed and an in-depth interview was conducted with a total of 22 individuals who are assumed to have better information on the issue, and a total of five key informants who were from the administrative unit were involved in the study.

### 2.6. Operational definition

➢ Occupational injury– any physical injury condition sustained by a worker in connection with the performance of their work in coffee processing industries.
➢ Work-related disease symptoms– symptoms of diseases that the workers developed during their stay in the coffee processing industries. In this study we have assessed the diseases' symptoms which include respiratory symptoms (cough, wheezing, and breathlessness) and musculoskeletal symptoms (lower back pain, shoulder and neck pains).
➢ Shoulder and neck pains–ache, pain, or discomfort felt at a time in the shoulder and neck (cervico-brachial region) in the last 6 months.
➢ Lower back pain–perceived self-reported pain and/or discomfort, localized between the coastal margin (bottom of the ribs) and above the inferior gluteal folds (top of the legs) which have lasted for days or weeks during the last 6 months.
➢ Respiratory symptoms–having at least one of cough, shortness of breath, or wheezing.
➢ Cough–participants were considered to have a cough at least if they cough first thing in the morning, cough during the day or night, cough as much as four to six times a day for a week, or cough on most days for as much as three consecutive months of the year.
➢ Work-related shortness of breath–participants were considered to be experiencing work-related shortness of breath if they usually experienced chest tightness while at work or just after work.
➢ Wheezing–participants were considered to be experiencing wheezing if their chest ever sounded wheezy (whistling sound).
➢ Hazards–a physical situation with a potential for human injury, damage to property, damage to the environment, or some combination of these.

### 2.7. Data collection and quality controls

#### 2.7.1. For quantitative study

The interviewer-administered structured questionnaire, which was adapted from similar studies was used (11, 19, 25–28). The disease symptoms mainly respiratory symptoms were assessed using the standardized questionnaire adopted from the American Thoracic Society (ATS) (29). In addition, musculoskeletal symptoms were assessed using the standardized Nordic questionnaire for the analysis of musculoskeletal symptoms (30). The questionnaire was translated to Amharic, and back-translated to English to ensure consistency. The data were collected by BSc. nurses and public health officers. Two days of training were given to data collectors about the objective of the study, the contents of the questionnaire, ways of interviews, and other related issues. A pretest was conducted on 5% of the samples in coffee processing industries that were not part of the actual study.

#### 2.7.2. For qualitative study

In-depth interviews and key informant interviews were conducted by two of the principal investigators using interview guides. The interviews were conducted in quiet places and responses were recorded by audio recorder.

### 2.8. Data entry, processing, and analysis

After checking for errors data were entered into Epi-data manager version 4.6.0.2 and exported to SPSS version 22 for analysis. The descriptive analysis was made using frequencies and proportions (percentages). Binary logistic regression was used to identify the association between the dependent and independent variables (work-related symptoms and occupational injuries). Variables with a *P* < 0.25 at bi-variable analysis were candidates for multivariable analysis. In multivariable logistic regression *p* < 0.05 was used to identify the significant factors affecting the outcome variables. Both the crude and adjusted odds ratios with the respective 95% confidence intervals were used to assess the strength of the association.

## 3. Results

In this study, multiple issues were addressed through a quantitative and qualitative approach. The qualitative study has focused on the experience of workers at coffee processing organizations mainly addressing issues related to awareness of occupational safety, utilization of personal protective equipment, the experience of work-related injuries, and experience of work-related musculoskeletal and respiratory diseases. Similarly, the quantitative study assessed and presented figurative data on the given issues. Details of the results are presented here below under different sections.

### 3.1. Socio-demographic and other general characteristics of the respondents

Out of a sample size of 842, a total of 721 workers participated in the quantitative studies, which makes a response rate of 85.6%. About half (48.1%) of the respondents were found in the age category of 19–29 years. The majority (60.2%) of the participants were male and about two third (64.9%) had attended primary education (Table 1).

**Table 1:** Socio-demographic and other general characteristics of the respondents, May/2021, Bench-Sheko and Kafa Zone.

**Variables**	**Categories**	**Frequency**	**Percent**
•Age in years	19–29	347	48.1
	30–39	258	35.8
	40–49	102	14.1
	≥50	14	1.9
•Sex	Male	434	60.2
	Female	287	39.8
•Monthly income in ETB	<1,000	152	21.1
	≥1,000	569	78.9
•Educational status	Can't read and write	81	11.2
	Can read and write	66	9.2
	Primary (Grade 1–8)	468	64.9
	Secondary (Grade 9-12)	106	14.7
•Marital status	Single	285	39.5
	Married	399	55.3
	Divorced and widowed	37	5.1
•Religion	Orthodox	330	45.8
	Protestant	293	40.6
	Muslim	73	10.1
	Others	25	3.5
•Ethnicity	Bench	135	18.7
	Sheko	137	19.0
	Amhara	58	8.0
	Oromo	61	8.5
	Kafa	315	43.7
	Other	15	2.1
•Employment status	Permanent	126	17.5
	Temporary	595	82.5
•Working location	Within the industry	258	35.8
	Out of the industry	328	45.5
	Both	135	18.7
•Service years in the industry	≤ 3 years	592	82.1
	>3 years	129	17.9
•Ever took job related safety and health training	Yes	81	11.2
	No	640	88.8
•Ever exposed to pesticide	Yes	29	4.0
	No	692	96.0
•Time of pesticide exposure	Within this year	20	2.8
	Before this year	9	1.2
•Working hour	Less or equals to 8	713	98.9
	More than 8	8	1.1
•Location of the kitchen	Inside the living room	98	13.6
	Outside the living room	623	86.4
•Fuel type used for cooking	Electric	1	0.1
	Wood	700	97.1
	Charcoal	20	2.8
•Body mass index in kg/m^2^	<18.5	35	4.9
	18.5–24.99	593	82.2
	>25	93	12.9

### 3.2. Occupational safety experience and behavioral characteristics of the respondents

All qualitative study participants were asked to define what occupational safety means and their experience regarding the occupational safety policy and related concepts. Accordingly, they have expressed as it does mean securing safety like protecting from injuries and exposure to risky conditions or reducing the risk of workers during their engagement in different given activities. Of course, they have described that they lack detailed information about the policy that focuses on such issues and can't mention any article or present a document. The key informants have reported as there is different health information dissemination to workers regarding occupational safety, how to preserve, and health and do works safely by following basic health protocols. The coffee processing organizations have clinics that serve the workers and it has also a referral system for advanced health problems.

#### 3.2.1. Utilization of personal protective equipment

All study participants have reported as personal protective materials are the most important things for any workers to facilitate activities, and reduce and avoid any risks to health and life. The most common materials reported to be used by workers include clothing, masks, gowns, gloves, and goggles. However, study participants have discussed as there is an interruption of material provision, quality problems, and inadequacy in terms of amount and timely replacement. Multiple things were mentioned to be the reason for the gaps in the area and mostly it was found to be due to under planning, budget problem, and improper utilization of the available materials.

#### 3.2.2. Occupational injury among study participants

**Figure 3:**
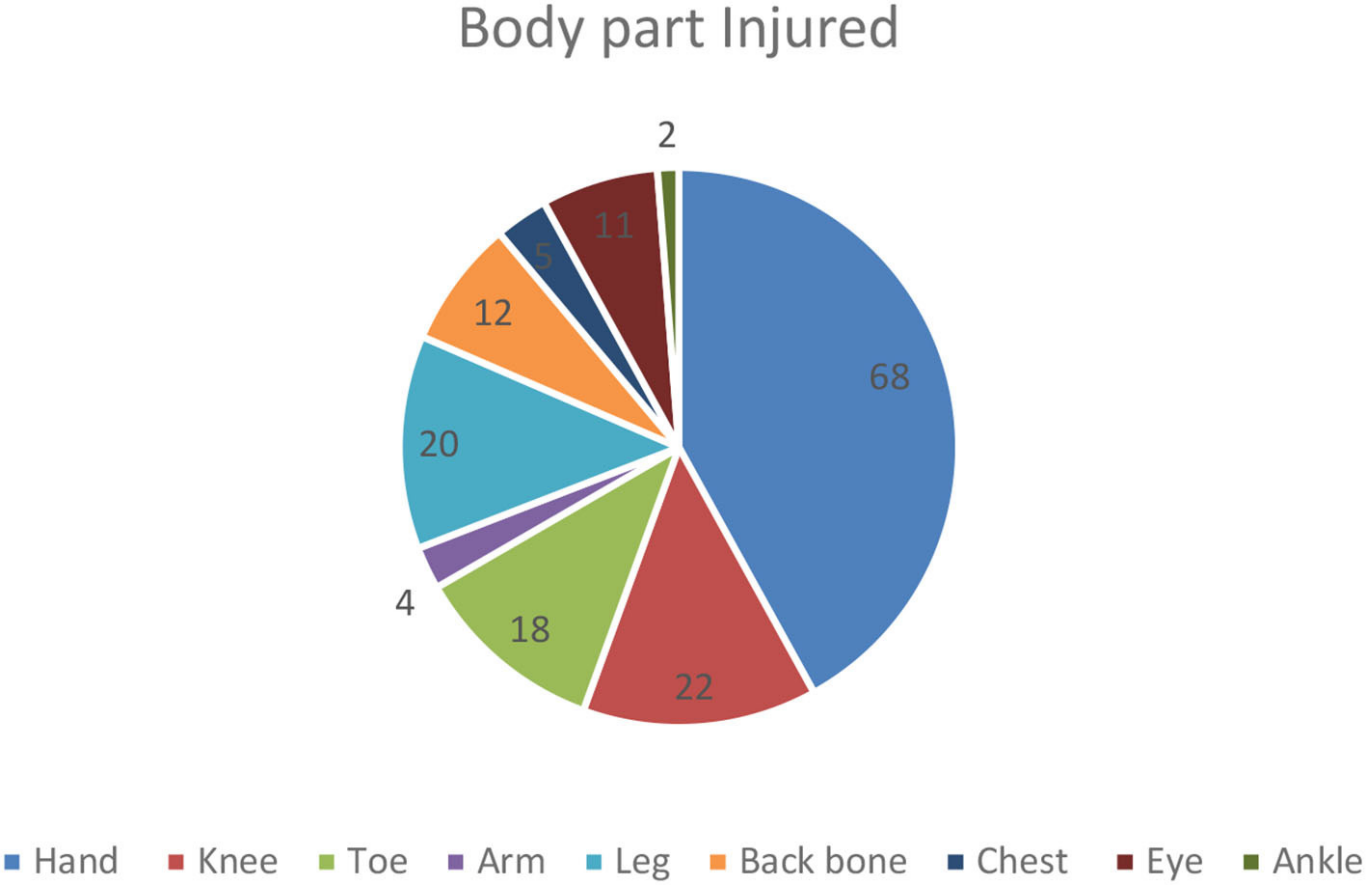
Body part injured among coffee industry workers, Bench-Sheko and Kafa zones, May/2021.

The coffee processing workers engage in different activities like lifting heavy loads, cutting different materials and wood, and collecting and packing the coffee. For doing that; they may use different sharp materials, machines, and chemicals and as a result, may face injuries. The following figure indicates the common kinds of bodily injury (Figure 3).

Through a qualitative approach; the most common kinds of injuries and health-related problems reported by participants include: machine hand cutting injury, pole injury, falling, snake bite, and back pain during carrying heavy loads. Many of them have been treated in the clinics and get recovered but sometimes the problem may end in complications, disability, and even death. The reasons for the occurrence of injury could be failing to take necessary care or lack of convenient working space and what makes the outcome poor could be late treatment, lack of adequate treatment, or irreversibility of the damage. It was also reported as there is no or enough compensation.

### 3.3. Work-related disease symptoms

Working environment and work conditions are among determining factors of health, and people may face different health problems while they are on their routine work. Workers from coffee processing industries included in this study have reported a history of respiratory diseases, occupational injuries, and different disease symptoms especially related to respiratory and musculoskeletal systems. The following tables indicate the most common types of health problems and disease symptoms (Tables 2, 3).

**Table 2:** Types of work-related health problems among workers in coffee industries, Bench-Sheko and Kafa Zones, May/2021.

**Types of health problems**	**Duration category**	**Frequency**	**Percent**
•Respiratory diseases	Less than a month	70	9.7
	At least 1 month	87	12.1
•Occupational injury	Less than a month	6	0.8
	At least 1 month	91	12.6

**Table 3:** Types of work related disease symptoms among workers in coffee industries, Bench-Sheko and Kafa Zones, May/2021.

**Type of symptoms**	**Frequency**	**Percent**
•Pain around neck and shoulder	114	15.8
•Lower back pain	353	49.0
•Cough	186	25.80
•Shortness of breath	66	9.2
•Wheeze	66	9.2
•Overall work related symptoms	398	55.2

Through a qualitative approach, the study participants have reported that the coffee processing area has much exposure to dust particles and pollens which may affect the respiratory system. People who are allergic might be sensitive and others could also develop certain health problems related to the breathing system. When asked if they ever have faced respiratory system-related diseases during their working period, some of them have reported repeated cough, sinusitis, wheezing, and breathing difficulty which challenges their life.

### 3.4. Factors associated with work-related disease symptoms and occupational injuries

During the bi-variable analysis, age, sex, income level, service year, working location, history of ever smoking, and personal protective equipment use were factors associated with work-related symptoms. However, age, income, service year, and history of smoking were independently associated factors. Likewise, age, income, service year, history of job-related training, pesticide exposure, personal protective equipment use, and current smoking status were associated with work-related (occupational) injuries during bi-variable analysis. However, only job-related training was significantly associated with occupational injuries. The details of bivariable and multi-variable analyses for both outcome variables were given in the following two tables (Tables 4, 5).

**Table 4:** Factors associated with work-related disease symptoms among workers in coffee industries, Bench-Sheko and Kafa Zones, May/2021.

**Variables**	**Variables' category**	**Work related symptoms**		**COR (95%CI)**	**AOR (95%CI)**	***P* value**
		**No**	**Yes**			
•**Age**	19–29	77	270	1	1	1
	30–39	39	219	1.86 (1.34, 2.59)	1.95 (1.37, 2.79)	0.000^*^
	40–49	23	79	2.62 (1.63, 4.19)	3.28 (1.89, 5.69)	0.000^*^
	≥50	4	10	0.63 (0.21, 1.93)	0.94 (0.27, 0.27)	3.274
•**Sex**	Male	177	257	1.50 (1.11, 2.03)	0.87 (0.60, 1.25)	0.441
	Female	146	141	1	1	1
•**Income in ETB**	<1,000	38	114	1.25 (0.17, 0.37)	1.24 (0.16, 0.36)	0.000^*^
	≥1,000	101	468	1	1	1
•Service year	≤ 3 years	86	506	1.27 (0.86, 1.85)	1.64 (1.04, 2.60)	0.034^*^
	>3 years	39	90	1	1	1
•**Working location**	Within the industry	103	419	1.42 (0.93, 2.16)	1.67 (1.00, 2.77)	0.051
	Out of the industry	18	134	1.02 (0.69, 1.53)	1.40 (0.86, 2.28)	0.173
	Both	11	36	1	1	1
•**PPE use**	Yes	68	127	1.76 (1.25, 2.47)	1.32 (0.88, 1.97)	0.177
	No	255	271	1	1	1
•**Ever smoking cigarette**	Yes	11	70	6.05 (3.15, 11.65)	5.59 (2.78, 11.26)	0.000^*^
	No	312	328	1	1	1

* Significant at p < 0.05.

**Table 5:** Factors associated with work related injuries, Bench-Sheko and Kafa Zones, May/2021.

**Variables**	**Variables' category**	**Occupational injury**		**COR (95%CI)**	**AOR (95%CI)**	***P* value**
		**Yes**	**No**			
•**Age**	19–29	70	277	1	1	1
	30–39	49	209	1.08 (0.72, 1.62)	1.25 (0.08, 19.45)	0.871
	40–49	34	68	0.51 (0.31, 0.82)	1.90 (0.12, 29.39)	0.646
	≥50	2	12	1.52 (0.33, 6.93)	2.69 (0.15, 49.81)	0.506
•**Income in ETB**	<1,000	43	109	0.62 (0.41, 0.94)	0.70 (0.11, 4.31)	0.696
	≥1,000	112	457	1	1	1
•Service year	≤ 3 years	106	486	1	1	1
	>3 years	49	80	0.36 (0.24, 0.54)	0.68 (0.20, 2.35)	0.543
•PPE use	Yes	74	121	0.30 (0.21, 0.43)	0.36 (0.11, 1.23)	0.102
	No	81	445	1	1	1
•Currently smoking cigarette^**^	Yes	16	36	2.60 (1.01, 6.70)	0.55 (0.17, 1.77)	0.315
	No	15	13	1	1	1
•Training related with job	Yes	30	51	1	1	1
	No	125	515	2.42 (1.48, 3.96)	11.88 (1.34, 105.57)	0.026^*^
•Pesticide exposure	Yes	13	16	1	1	1
	No	142	550	3.15 (1.48, 6.69)	1.05 (0.10, 10.80)	0.969

* Significant at p <0.05.

** Since all the participants were not smoking currently, four cells will not add up to 721.

## 4. Discussion

In our study, the overall prevalence rate of respiratory symptoms among coffee processing workers was 21.7%. The result is consistent with other studies done in Tanzania, Ethiopia, Papua New Guinea, and Uganda (31–33). All of these studies revealed that coffee workers have a high prevalence of respiratory health problems. However, our present study found a higher prevalence of some of the respiratory symptoms (such as cough 25.8%) compared with the studies done among coffee industry workers in Tanzania, Uganda, and Ethiopia (28, 34). This might be due to the higher personal total dust exposure in southwest Ethiopia's coffee processing industries. The different methods of coffee pre-processing might be another reason (4, 28). One of the study participants depicted the issues as follows:

“*I have repeatedly experienced common cold and breathing problem and I think it is due to the waste around the working area like accumulation of garbage and liquid waste…and sometimes I visit the clinic, take treatment and sometimes use home treatments…”*

Occupational safety and health problems are becoming major challenges in Ethiopia because of low occupational hazard awareness, lack of factory safety and health policy, and inefficient safety management systems (24). In our study, the overall occupational injuries among coffee processing industry workers were 13.4%. Work-related injuries were significant problems for the coffee processing industry workers. A total of 353 (49%) workers experienced low back pain during the last year, and 114 (15.8%) faced pain around the neck and shoulder during their current job. This work-related pain was considered to be severe enough for most workers to seek medical attention or take days off. Moreover, study participants in coffee processing industries substantiate this finding and explained the issues as follows:

“*My friend was hand broken, His fracture was due to falling by sliding and he was treated traditionally and healed after he got sick leave with pay. But there was no additional support given for him.”*

“*I was fallen earlier when washing coffee during harvesting time, due to sliding and was sick for 6 months having no sick leave without payment, and at that time I was not able to feed my family. The company did even not cover my treatment expense. The reason for my falling is, due to lack of necessary protective equipment and I was unable to overcome the hardship of the heavy work even not eaten well.”*

“*Cutting wounds by sharp equipment and machines up to death rarely, falling, fractures, disability; possible reasons for the accidental injuries are not following the necessary self-care during using long ladders that ends in falling. Lack of sufficient PPE, due to this, there are several workers walk by crutches ad suffer from chronic back pains.”*

These findings are lower than other studies conducted in small-scale industries in Nigeria and Ethiopia (4, 12, 28). This might be due to seasonal variation and the working condition of the industries.

In the current study, age, income, service year, and smoking cigarettes were significantly associated with work-related symptoms. Coffee processing industry workers who have age group 30–39 and 40–49 were 1.95 and 3.28 times more likely to have work-related disease symptoms respectively. This could be attributable to workers who have children might spend more time in the routine activities and coffee processing area and face more exposure to occupational hazards (4).

Besides, coffee processing industry workers who have a monthly income of <10,000 ETB were 1.24 times more likely to have occupational-related disease symptoms. This might be due to workers who have less income could be unable to purchase personal protective equipment. As a result, working without using complete body covering PPE could expose the worker's body to different kinds of occupational hazards. This study is comparable with other studies done in Tanzania, Ethiopia, Papua New Guinea, and Uganda on coffee processing industry workers (27, 31, 32).

Coffee processing industry workers who worked less or equal to 3 years were 1.64 times more likely to have work-related disease symptoms. The reason for being exposed to this level relates directly to the length of time workers spent on coffee processing activities. Similarly, studies done in Uganda and Tanzania (3, 4) showed that working experience was significantly associated with work-related symptoms among coffee processing industry workers. In addition, another study done in Ethiopia showed that work experience was significantly associated with occupational health conditions (11, 13, 25). Another possible reason for exposure to occupational health risks might be staying for a long time as coffee processing workers, which increases exposure to determinant factors.

Furthermore, coffee processing industry workers who smoke cigarettes were 5.59 times more likely to have work-related disease symptoms compared to their counterparts. This result contradicts other studies done in Ethiopia and Tanzania in which smoking cigarettes was not significantly associated with a respiratory infection and work-related symptoms (19, 27, 28, 34). This might be the smoking habit of the study participants. In addition, coffee processing industry workers who did not take training related to jobs were 11.88 times more likely to have occupational injuries compared with their counterparts. This finding was higher than a similar study conducted in Papua New Guinea (35).

## 5. Conclusion

The prevalence of work-related symptoms and occupational injuries is high among coffee processing industry workers in southwest Ethiopia. Some socio-demographic and workplace factors, which include age, income, service year, and smoking cigarettes, were found to be significantly associated with work-related disease symptoms. In addition, training related to the job has also been significantly associated with work-related injuries. Hence, it is important to provide appropriate and full body cover personal protective equipment with adequate training and manage the working hours. Moreover, there is a need for regulations at both government and private (factory owners) levels to improve the working conditions and ergonomic structure of coffee processing industries. Besides, it is important to have further studies to quantify the ergonomic hazards, especially chemical and dust level exposure, are recommended.

## Data availability statement

The raw data supporting the conclusions of this article will be made available by the authors, without undue reservation.

## Ethics statement

The studies involving human participants were reviewed and approved by Mizan-Tepi University College of Medicine and Health Sciences Research Ethical Review Committe. The patients/participants provided their written informed consent to participate in this study.

## Author contributions

BM, NS, and WW have analyzed the data and prepared the manuscript. MT and SN have reviewed the manuscript. All authors have participated in writing the protocol and data collection and read and approved the final manuscript.
